# Analysis of actors’ attitudes towards the application of the legal institutional framework for sand mining in two local government areas of Ghana

**DOI:** 10.1371/journal.pone.0355691

**Published:** 2026-08-13

**Authors:** Kofi Yeboah Asare, John Victor Mensah, Joseph Boateng Agyenim, Emmanuel Yamoah Tenkorang

**Affiliations:** 1 Department of Development Management, Institute of Local Government Studies, Ogbojo, Accra, Ghana; 2 Department of Integrated Development Studies, School for Development Studies, University of Cape Coast, Cape Coast, Ghana; Cranfield University, UNITED KINGDOM OF GREAT BRITAIN AND NORTHERN IRELAND

## Abstract

Sand is illegally mined in Ghana, despite the existence of legal frameworks governing the industry. The paper analyses actors’ attitudes toward the application of the legal institutional framework for sand mining, using the Ga South and Gomoa East local government areas as a case study. Qualitative interviews and documentary analysis were employed. Data were collected through qualitative interviews with 32 sand-mining stakeholders, Focus Group Discussions (FGDs) with women and youth groups, and documentary analysis. NVivo 12 software was used for thematic and content analysis. The results revealed that certain aspects of the legal frameworks related to licences, compensation for affected parties, complaint channels, and stakeholder participation were poorly enforced. This failure is linked to insufficient regulatory staff for regular compliance monitoring, outdated monitoring systems, poor coordination among regulatory agencies, rent-seeking, and political patronage. We recommend that the sector ministry initiate a consultative governance system by establishing a coordinating secretariat to harmonise regulatory efforts among all stakeholders. At the same time, laws on sand mining should be consolidated into a single policy document. The central government should also adequately resource the regulatory agencies with advanced monitoring equipment.

## Introduction

Sand is a vital extractive resource for the rapidly urbanising world because of its connection to infrastructure development, economic growth, and job creation for both skilled and unskilled workers [1–4]. Sand mining also contributes to the production of fracking technology, computer chips, glass, paints, and beach nourishment, among other applications [5–7]. Despite its widespread use, conversations about mining focus on the trade of precious metals (gold, bauxite, and diamonds) and energy minerals (fossil fuels) because they have the potential to generate foreign exchange [1]. Although not often highlighted, sand is the largest mined resource by volume, totalling 50 billion metric tonnes annually [8,9].

As is characteristic of the extractive industry, the fundamental discourse on sand mining in the Global South focuses predominantly on illegal extraction and adverse externalities [9–14]. Unregulated sand mining contributes to widespread environmental degradation, disruption of local livelihoods, loss of soil fertility, food insecurity, pollution, conflicts, abuse of weaker actors, and depletion of forests, among other consequences [4,11,15–17]. Drawing on Social-Ecological Systems (SES) theory, Preiser et al. (2018 [18]) argued that human survival, including material needs, personal safety, good social relations, and freedom of choice, relies entirely on the biosphere’s capacity and its interaction with the Earth system through existing natural resource regimes. Unsustainable sand mining is a potential threat to the biosphere and to the attainment of the Sustainable Development Goals (SDGs) [9,10,14]. It is often prompted by inadequate governance or a lack of enforcement of existing mining laws to protect the environment and vulnerable actors [5,9,19,20]. In the absence of a well-defined regulatory framework and in the presence of systematic power asymmetries, influential actors often engage in resource capture and environmental extractivism, culminating in unsustainable outcomes [21,22].

Governance of sand mining is embedded in a complex interaction between social and ecological systems, encompassing individual, community, and social institutions, cultural values, and belief systems [23]. Though sand is not a common-pool resource in Ghana, the sand mining industry comprises a complex assemblage of actors with diverse interests. Consistent with Ostrom’s (2010 [24]) polycentric governance theory, there are three complex governance settings – the private sector, the government sector, and the third sector – for sand mining in Ghana [25]. The private sector (market governance) actors undertake the actual extraction, and they mostly fail to ensure sustainable outcomes [11,17,26]. The government sector (state governance) is required to implement interventions, including establishing and enforcing legal frameworks to address market failures and pursuing policies that balance economic growth and environmental stewardship [25]. Ostrom explained, from polycentric governance theory, that the primary causes of governance lie not in actors’ choices but in laws, practices, and underlying social orders. Because individuals make their choices under conditions of ‘bounded rationality’, formal institutions play a central role in shaping behaviour. The argument goes further beyond market and state governance, recognising reforms that empower civil society organisations (community governance) to foster accountability and environmental quality [24]. Ostrom hypothesised that the bottom-up approach to natural resources governance, involving relevant public, private, and civil society actors, provides the highest societal welfare and sustainability. The multiple autonomous decision-making centres and actors involved in sand mining include sand contractors, landowners (chiefs, family or clan heads), truck drivers, local government bodies, and regulatory agencies such as the Minerals Commission and the Environmental Protection Agency (EPA).

In Ghana, sand is classified as an industrial mineral and is governed by the Mineral and Mining Act, 2006 (Act 703), as amended in 2010, 2015, and 2019, and by the Environmental Assessment Regulations, 1999. Apart from these laws, the Local Governance Act, 2016 (Act 936) empowers local government authorities to formulate bylaws to regulate mining activities within their jurisdiction. Ghana’s mining laws vest the right to extract mineral resources in the President. Sections 76–80 of the Minerals and Mining Act 703 are dedicated to industrial minerals, addressing issues including licensing regimes, reconnaissance, and non-citizen rights to industrial mineral rights, among others. Other issues in the laws, including environmental rehabilitation, the composition and functions of the district mining office, obligations of miners, and land tenure arrangements, are encapsulated in sections 82–99 of Act 703.

**Fig 1:**
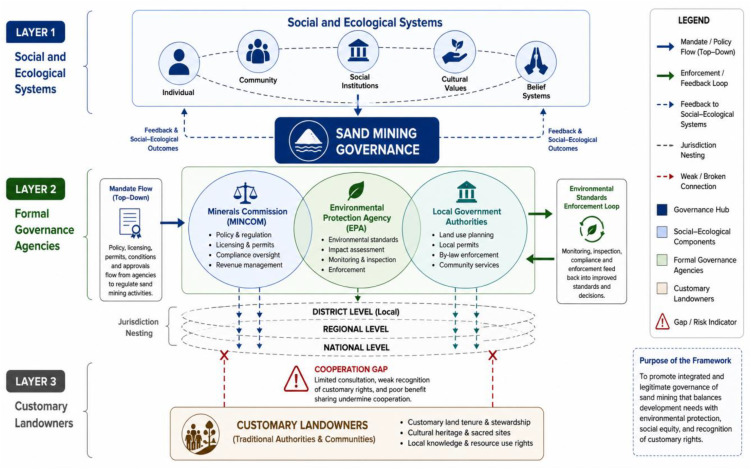
Ghana’s sand mining governance framework and the institutional linkages between responsible agencies.

The implementation of the legal governance framework for sand mining falls under the jurisdiction of the Minerals Commission (MINCOM), the Environmental Protection Agency (EPA), and local government authorities (Fig 1). These agencies are mandated to issue licences and permits and to monitor sand miners to ensure that environmental standards are maintained. The governance arrangements are often described as overlapping because they are nested across multiple jurisdictional levels (for example, district, regional, or national) and cut across different regulatory agencies and institutions. The varied interests and concerns of the diverse actors involved in sand mining must be addressed to attain sustainable governance [24].

Despite the availability of a legal framework for mining and multiple agencies responsible for ensuring sustainability in the sector, over 80% of sand mining in Ghana is done illegally [11,17,27]. The widespread illegal practices characterising sand mining raise concerns about the efficacy of the existing legal regulatory frameworks [11,14]. Sustainable sand mining requires a legal and regulatory governance system that balances the need for economic development with environmental conservation and social well-being [28]. Though several scholars have identified the socio-environmental outcomes of sand mining [8,20,26,29–31], the legal context for addressing its environmental ramifications has not been adequately examined. There is a paucity of information on the governance challenges inherent in the sand mining sector of Ghana, amidst the widespread illegal extraction. Therefore, using the Gomoa East District and Ga South Municipality, which are known for sand mining, as a case study, this paper sought to build on the existing literature to analyse the attitudes of sand mining actors towards the implementation of the legal institutional framework for sand mining at the subnational level, using the lens of polycentric governance theory. The paper addresses two key research questions. First, how is the existing legal institutional framework for sand mining applied in the mining host communities? Second, what bottlenecks do regulatory agencies face in ensuring compliance with Ghana’s sand mining laws?

The paper contributes to the understanding of the governance challenges of sand mining. It aims to promote a collaborative governance system that emphasises shared interests and aligns the efforts of diverse actors in the sand value chain to ensure compliance with regulatory requirements and sustainability. The study also provides lessons for other local government areas and regions affected by illegal sand mining. Following this introduction, the paper is divided into five parts: materials and methods, results, discussions, conclusions, and recommendations.

## Materials and methods

The qualitative case study research design, based on the interpretivist paradigm, was the ideal approach for the study. The qualitative design offers flexibility to explore respondents’ experiences, allowing them to present reality from their perspectives [32]. The study was undertaken in Tebu and Hobor, located in the Ga South municipality, and Budutatta and Kweikrom, also in the Gomoa East district of Ghana, which have been historically known for widespread illegal sand mining over the past 30 years (Key Informant at MINCOM, 2022). The Ga South Municipality lies within latitudes 5048’N and 5029’N and longitudes 008’W and 0030’W, occupying a land area of about 358 sq km. The majority of residents in the municipality are farmers who cultivate crops such as maize, groundnuts, cassava, cowpeas, pineapples, mangoes, and cashews. The Gomoa East district is located within latitude 5° 31’ 59.99” N and longitude 0° 24’ 59.99” E, occupying a total surface area of about 276.652 sq km. Similarly, the majority of the residents in the local government area are employed in land-based livelihoods, especially farming. The selected study sites constituted an ideal case for three main reasons. First, the areas are well endowed with the desired sand for the building industry. It is estimated that 4.55 million m3 of sand is mined from these areas annually [11]. Second, the areas are close to Accra, which has a very high demand for sand due to the rapid urbanisation. Third, the areas also offer comparatively lower transport costs for the conveyance of sand to Accra, which is about 29 km and 42 km away.

The study population comprised actors involved in diverse activities within the sand mining sector, including local chiefs/landowners, sand contractors, community development committee members, tipper truck drivers, and state officials of the EPA, the MINCOM, local government authorities, and the Department of Agriculture. The actors involved in sand mining are homogeneous; thus, the population of the study areas is similar to other sand mining communities in Ghana [11,33].

The purposive sampling technique was used to select officials of the EPA, the Minerals Commission, local government authorities, the Department of Agriculture, and landowners, with their knowledge of sand mining as the selection criterion. On the other hand, the accidental sampling technique was used to select the sand miners and tipper truck drivers due to the nomadic nature of their operations. A total of 32 key informants were interviewed because further interviews were not generating new information, suggesting data saturation (Table 1). Data saturation is a point in data collection and analysis at which new data or information no longer contributes significantly to addressing the research objective, or when existing data is replicated [34,35]. Hennink and Kaiser (2022 [36]) proposed that a sample size of 12–15 respondents in a relatively homogeneous population is adequate to achieve saturation. In addition to the key informants, two FGD sessions, comprising 7–12 persons, were organised for members of both youth and women’s associations in each of the communities. This was done because, in Ghana, the majority of sand-mining actors are males over 40 years old [11,17]. Therefore, it was important to capture the realities of youth and women. Additionally, employing multiple data collection methods provides a richer description of the phenomenon under study and enables faster data saturation [36,37].

**Table 1:** List of key informants interviewed.

Actor	No. interviewed	Sampling procedure	Percentage
Landowners/chiefs	8	Purposive	23.5
Sand contractors/miners	8	Accidental	23.5
Tipper truck drivers	7	Accidental	20.6
Community development committees	2	Purposive	5.9
Key informant at the local government authority	2	Purposive	5.9
Key informant at the Department of Agriculture	2	Purposive	5.9
EPA	2	Purposive	5.9
MINCOM	1	Purposive	2.9
Total	32		100.00

Source: Field survey, 2021

Both secondary and primary sources of data were utilised. Interviewing, FGDs, and documentary analysis were the data collection methods employed. The key informant interviews were recorded after soliciting the respondents’ consent. Participants were first asked questions about their demographic characteristics and experiences. Afterward, they were asked to identify the legal framework for sand mining and how their agencies implemented it. Additional questions centred on regulatory challenges, the nature of collaboration among the stakeholders, sanctions for illegal mining, and suggestions for sustainable governance of sand mining. Follow-up questions were asked during the interviews to clarify some specific issues.

The documentary review examined the Minerals and Mining Act 703 and the Environmental Assessment Regulations of 1999 to identify themes for the study. Documentary review is an ideal approach for assessing regulations, policies, and other documents relevant to environmental resource governance because it helps to uncover hidden meanings and themes that are not evident through interviews [38]. The documentary review facilitated the identification and selection of key informants knowledgeable about sand mining governance. Additionally, the documentary review served as a triangulation tool to confirm the findings from the primary data. The data collection exercise commenced on March 3, 2021, and continued until May 20, 2021. Additional data were collected from the 10th to the 21st of January 2022 through selected key informants to clarify some responses.

The responses from the key informant interviews, FGDs, and thematic analysis were cleaned, transcribed, reduced, and discussed to extract qualitative information. INvivo version 12 was used to analyse the data using thematic content analysis. This involved systematic examination, analysis, and reporting of the key themes within the dataset [39]. A total of 57 sets of themes were identified in the data analysis process, with repetitive themes emerging and no new relationships between the themes, thus signifying attainment of thematic saturation. A Strengths, Weaknesses, Opportunities, and Threats (SWOT) analysis was also done for the formal regulatory agencies.

The research was conducted in conformity with ethical standards in social science research to ensure confidentiality and anonymity of responses. Ethical clearance number UCCIRB/CHLS/2020/48 was obtained from the University of Cape Coast Institutional Review Board (UCC-IRB) before fieldwork. A written informed consent letter was issued to the EPA, local government bodies, and MINCOM to explain the purpose of the research and solicit their voluntary participation. For the other respondents, including landowners, truck drivers, sand contractors, community development committee members, and FGD participants, copies of the written consent statement were issued and explained to each of them. Each participant endorsed the consent form to indicate willingness to participate before the interview or FGDs commenced. All respondents were informed that they had the right to withdraw from the study at any time. Respondents and their information were treated with strict confidentiality and anonymity, maintained through data coding that made individual responses impossible to isolate. In addition, the authors did not have information that could identify individual survey participants after data collection.

## Results

The analysis focused on the thematic areas that emerged from the documentary review, including allocation of land for sand mining, permits and licences, stakeholders’ involvement, compensation and remedies to adversely affected persons, and the availability of platforms for addressing complaints against sand miners. The challenges confronting the regulatory agencies in ensuring compliance with mining laws were also examined.

### Land acquisition for sand mining

The interactions with the key informants revealed that the landownership system of the study communities was customary, consistent with most parts of Ghana, where obligations to customary land are vested in lineages represented by chiefs, traditional priests, family or clan heads. It was evident in Hobor and Tebu (migrant communities) that, while the majority of residents were Ewes, the land was administered by the Ga clan heads, who were the allodial titleholders. The landowners in Hobor and Tebu reported that the Ga people followed patrilineal inheritance, so land was inherited through male members of successive generations. Therefore, the settler residents had access to land in two ways. First, most of the settlers depended on undocumented tenancy agreements with allodial title holders for their land-based livelihoods. This approach was embedded with several insecurities, and aggrieved tenants could not seek legal redress. Second, a few settlers had purchased land from the traditional landowners. In Buduatta and Kweikrom, family heads and chiefs controlled the redistribution of land to the community members.

The mining laws underscore that all minerals in their natural states on or under the surface of the soil are vested in the state. However, the laws allow landowners to exercise surface rights such as farming, fishing, or forest gathering in the interest of their welfare. Participants revealed persistent wrongful allocation of land by traditional leaders, including chiefs and clan or family heads, for sand mining without authorisation from regulatory agencies. Key informants at the local government authorities and MINCOM emphasised that landowners refused to accept the law regarding state ownership of minerals. The contentious position had created an agency for the traditional rulers to manoeuvre the formal regulatory actors to engage in sand mining on the blind side of the state. The lack of understanding and acceptance of the difference between mineral rights held by the state on one hand and surface rights of landowners on the other was confirmed by a landowner in these words:

*As it stands, most landowners believe that while the lands belong to them, regulatory agencies accrue gains from sand mining. Therefore, some landowners allocate land and collaborate with illegal sand miners to enjoy financial benefits (Landowner in Gomoa Buduatta, 2021).*

The documentary review highlighted that the sand contractors must first obtain the landowner’s approval, after which the parties must agree on the types and forms of compensation for the designated land. However, the interactions revealed that the informal agreement between the landowner and sand contractor was erroneously perceived as legal permission to extract sand, thereby leading to widespread unlicensed mining.

### Mining licences and permits

The key informants at the MINCOM, EPA, and local government authorities explained that to undertake sand mining in a designated area, sand contractors were required to acquire licences and permits through a formal application process at the MINCOM, EPA, and local government agencies. The licence and the environmental permit, as detailed in Act 703 and the Environmental Assessment Regulations (LI 1652), were subject to a systematic evaluation of the objectives and alternatives for the proposed mining undertaking, as well as its expected impacts on the environment and the host communities. Key informants at the regulatory agencies highlighted that the minister for the Ministry of Lands and Natural Resources was the sole authority permitted by law to approve restricted mining leases.

Most sand contractors operated without a licence or used a single licence to mine beyond the demarcations of their licences, in contravention of the Minerals and Mining Act 703. The participants expressed shared concerns about the phenomenon, noting that it impedes the attainment of sustainable governance in Ghana’s sand mining industry. Some participants attributed the widespread absence of permits and licences to the culture of infrequent compliance monitoring and enforcement by the MINCOM and the EPA. Officials at the EPA and MINCOM blamed the situation on the ‘moral hazard’ problem of the miners and the low penalty regime for illegal sand mining. A key informant at the EPA shared these words:

*The sanction for sand mining offenses under Section 29 of the Environmental Assessment Regulation, 1999 (LI 1652) is GHS 200 ($20). It is more economically rational for sand miners to break the rule than to comply with it. The penalty should be adjusted upward to deter miners from such practices (Key informant, 2021).*

It can be inferred from the statement that, because the sanction for noncompliance was relatively low-priced, illegal sand miners preferred the penalties to the permit acquisition. The EPA official raised concerns regarding the outdated sanction regime in the Environmental Assessment Regulation of 1999, calling for a review of the noncompliance sanctions to serve as a deterrent to illegal sand miners.

The finding also reveals the bureaucratic process for obtaining sand mining licences from the MINCOM, the EPA, and local government agencies, which discourages sand miners from following the formal process. The delay in processing sand mining permits was a breach of Act 703. The process was bureaucratic and associated with both formal and informal costs. Some of the sand contractors reported that the formal application process was a complete waste of time. However, the regulatory officials emphasized that the licensing process takes no more than three months if sand contractors meet all requirements. From the perspective of the regulatory actors, three key issues emerged as constraints to the timely processing of permits and licences. First, the applications from the sand contractors often had several lapses and lacked the requisite supporting documents. Second, multiple agencies are involved in the license/permit process, necessitating inter-agency consultations. Third, the occasional non-availability of the sector minister, who signs all licences on behalf of the president.

As part of the permitting process, sand contractors were required to register their businesses with the Registrar General’s Department as a prerequisite for approval of a mining license. However, some sand contractors admitted they had not registered their businesses. The interactions revealed that, although the situation was not widespread, it could worsen if left uncontrolled. The situation was attributed to interference by some government officials who use their powerful positions to influence licensing decisions in favor of their friends and cronies. The interactions established that, because the mining laws accorded the sector minister overall authority to grant licenses to prospective miners, the minister could ignore MINCOM’s advice and instruct the commission to process licenses for unqualified individuals as a result of political patronage. A key informant remarked:

*Some of the sand miners are well-connected to ‘big men’ in authority. It is very difficult to reject the applications of these miners due to the influence of their powerful friends. We cannot sanction these categories of sand miners even when they engage in illegal extractions (Minerals Commission, 2021).*

### Stakeholder involvement

The mining laws of Ghana highlight the importance of stakeholder involvement and collaboration in fostering efficient governance of the extractive industry. Act 703 makes chiefs integral members of District Mining Committees. However, participants—especially landowners and Community Development Committee members—raised concerns that formal regulatory actors allegedly disregarded them in licensing, permitting, monitoring, and enforcement. A landowner shared these words, stressing the marginalisation of informal actors in the governance of sand mining:

*The state agencies expect us to monitor the activities of sand miners, but they have marginalised landowners in the licensing process. How can we support them if the relationship between us is poor? They have to acknowledge that we owe the land and also have better knowledge of the local terrain (Landowner in Tebu, 2021).*

For instance, the MINCOM is required to collaborate with local government authorities and officially publish copies of the application for a sand mining licence in the designated mining host communities and in public newspapers. The publication was intended to give landowners and other residents adequate notice of the proposed mining undertaking and to provide an avenue for potential public agitation. There was a convergence of ideas among landowners and FGD participants in the study areas, noting that they had never come across such notices in their communities. The key person at MINCOM noted that the commission relied solely on newspaper publication and shared these words:

*The Commission previously sent all proposed applications to the local government bodies for posting in the sand mining communities. However, the local authorities are not cooperative and often fail to publish the applications. All they care about is using the advert as an avenue to generate revenue from the sand miners. We have decided to use newspaper publications instead of relying on them.*

The intrinsic challenge of using newspapers to disseminate such important information in rural and peri-urban areas of Ghana, where illiteracy is relatively high, is that many people may lack access to it. Participants reported that the information gap and residents’ non-involvement in mining decisions often resulted in their unanticipated displacement from their farms, leading to livelihood insecurity. Besides, the inadequate involvement of informal actors was cited as a key reason for land-use disputes embedded in sand mining. Participants in the study areas corroborated insights from polycentric governance theory that effective involvement of local stakeholders, including chiefs, family or clan heads, and community members, in sand mining governance could lead to more efficient sector governance. FGD participants explained that a polycentric governance approach would forestall accountability and trust among actors, facilitate the smooth flow of information, deepen inclusivity, and reduce high levels of violence characterising the sand mining industry.

### Compensation/ Remedies for negatively affected people

The documentary review of Act 703 revealed that holders of mineral rights, including sand contractors, are required to compensate the landowners for the deprivation of their surface rights to the land. Besides, adversely affected residents should be compensated for loss or damage to immovable properties, loss of potential earnings from alternative land use, and crop life expectancy benefits where the prospective mining areas are cultivated. Participants in all the study areas revealed that aside from the landowners, other residents who were adversely affected by sand mining in the form of damage to buildings, crops, or cropland were often not compensated by the miners. On a few occasions, some adversely affected residents received meagre cash compensation from the miners. However, the compensation amounts were unilaterally determined by the sand contractors and therefore were not proportional to the damage to the properties.

It was evident that, contrary to Act 703, the Land Valuation Department of the Lands Commission was often not consulted when determining fair compensation for residents who lost their farms, crops, or other properties. A key informant of the Department of Agriculture remarked:

*The sand contractors solely decide how much to pay as compensation to the affected farmers. As a result, the meagre compensation often ranged from GHS 150 to GHS 450 ($15 to $45) in total, irrespective of the size of the farm or crops destroyed. Sometimes the Department of Agriculture is consulted by the local police to assess the value of the crops destroyed, but farmers often complain that our recommendations are not used.*

The narrative suggests that the regulatory agencies failed to assist negatively affected residents in obtaining commensurate compensation for the destruction of their properties caused by the sand miners. The finding underscores that the lack of land rights or formal tenancy agreements to farmland, especially among settler residents, was a major hindrance to the right to compensation.

### Availability of complaint platforms

Participants revealed that most had inadequate access to complaint channels for addressing issues with sand miners. They noted that residents reported sand-mining-related complaints to the local police, local government authorities, chiefs/landowners, spiritual leaders, Assembly Members, and/or community development committees. None cited the EPA or MINCOM as avenues for addressing sand mining complaints because their administrative structures were in distant locations outside the jurisdiction of the studied local government areas at the time of the study.

Two key challenges with the aforementioned complaint platforms were reported. First, the compliant platforms were less favourable to settler residents than to locals who were indigenes of the study areas. Indigenous residents had an option to complain to the allodial title holders of the land (chiefs or clan heads), while the settlers had limited relations with the landowners. Besides, the majority of the settlers had no formal agreement governing the use of their farmland; therefore, they rarely reported issues related to displacement from their farms to the local police. The second issue mentioned was the ineffectiveness of the complaint platforms. Some participants alleged that the local police personnel demanded money from adversely affected residents before listening to their complaints. Some of the participants also reported that the local government authorities did not follow up on cases reported to them. Others cited repeated follow-ups to the regulatory authorities, but often to no avail.

However, key informants at regulatory agencies reported three reasons for the ineffectiveness of sand mining-compliant channels. First, the agencies lacked adequate human and financial resources to investigate all the complaints they received regarding illegal sand miners. Second, excessive delays by locals in reporting complaints against miners made it difficult for the agencies to follow up on the issue, since the miners operated in a migratory manner. Third, the unwillingness of residents to volunteer information about illegal miners to aid regulators’ investigations. Participants at the local government agencies highlighted that the security councils of local authorities occasionally conducted special enforcement exercises to address complaints related to illegal sand mining. It was revealed that the security council sometimes arrests illegal sand miners and also impounds their mining machinery. However, none of the local government authorities had embarked on such special enforcement exercises for the past seven months as of the time of the data collection. A key informant of the Department of Agriculture shared these words:

*It is the responsibility of the local government authority to ensure that the environment is safe for both humans and nonhumans and to promote the livelihoods and socio-cultural needs of the residents. At present, the sand mining situation is very widespread and difficult to regulate. As a result, the complaints are equally overwhelming for the authorities to handle.*

It is inferred from the above narrative that the regulatory agencies have not met the expectations of the local people regarding the provision of a channel to address their challenges with sand mining effectively. Therefore, safeguarding the environment and the livelihoods of residents in sand mining communities was not pursued, as residents were left to their fate to battle the widespread, unsustainable sand mining.

### Challenges confronting the regulatory agencies

The interactions with key informants highlighted the governance challenges that hinder regulatory agencies from effectively regulating sand mining in Ghana. In this section, we present the bottlenecks that obstruct regulatory agencies from effectively implementing the legal framework. Strengths, weaknesses, opportunities, and threats (SWOT) analysis, as shown in Table 2, was utilised based on the key informant interviews.

**Table 2:** Strengths, Weaknesses, Opportunities, and Threats (SWOT) matrix for sand mining regulator.

Agency	Strengths	Weaknesses	Opportunities	Threats
EPA	• Existing laws on the environment • Legal backing • Qualified staff	• Limited staff • Inadequate logistics • Low collaboration • Low monitoring • Information asymmetry on sand mining • Absence of state-of-the-art technology	• Global recognition (SDGs) •Collaboration with other agencies (logistics, joint monitoring)	• Urbanization • Political/big men interference • Assault by illegal miners • Dispersed mining locations
Minerals Commission	• Existing laws on mining • Legal backing • Qualified staff	• Limited staff • Inadequate logistics • Low collaboration • Bureaucracy • Low monitoring • Information asymmetry on sand mining • Absence of state-of-the-art technology	• Media attention • Collaboration with other agencies (logistics, joint monitoring)	• Political/big men interference • Assault by illegal miners • Dispersed mining locations
MMDAs	• Legal backing • Proximity to mining site • Close association/ representation in the communities	• Inadequate logistics • Low collaboration • Lack of bye-laws • Low monitoring	• Media attention • Collaboration with other agencies (logistics, joint monitoring)	• Assault by illegal miners • Urbanization • Political interference

Source: Fieldwork, 2021

#### Inadequate inter-agency collaboration.

The participants reported that the implementation of the mining laws was fragmented among several agencies, with inadequate collaboration, which manifested in the licensing and permit processes, a lack of joint monitoring and enforcement exercises, irregular inter-agency meetings, and limited information sharing. For example, key informants at local government agencies alleged that MINCOM granted mining permits to sand contractors without recourse to the local authorities’ land use and spatial planning schemes. It was gathered from the interactions that inadequate information sharing, coupled with complexities and overlapping functions of the regulators, had led to inter-agency conflicts, which adversely affect the efficient governance of the sand mining industry. The key informant at the MINCOM confirmed the finding in these words:

*The lack of collaboration had created inter-institutional conflicts and suspicion among the regulatory agencies. This manifested in a ‘blame game’ syndrome where officials in different agencies see the others as the cause of the problems characterising sand mining.*

The comment affirms that the absence of collaboration among the regulators is a major hindrance to the sustainable management of sand mining. The regulators stressed the importance of establishing a coordinating secretariat to harmonise the efforts of the various administrative agencies and units, ensuring adequate collaboration for the sustainable governance of sand mining.

#### Absence of advanced technology and logistics, and inadequate personnel.

The widespread sand mining activities in the study areas were carried out in a migratory manner from dispersed locations, thus requiring sufficient logistics, advanced technology, and adequate personnel to effectively manage the sector. However, a consistent theme emerged regarding the lack of state-of-the-art technology and logistics, such as remote sensing or satellite imagery facilities, drones, and vehicles, to detect ongoing illegal sand mining in real-time. For instance, key informants at both the MINCOM and the EPA highlighted that their agencies did not embark on monitoring and enforcement exercises at night due to the risk involved. The study underscores that adopting advanced technology could help ensure efficient 24-hour monitoring, safeguard the environment, enhance productivity, reduce costs, and collect data across all sand mining sites. Participants emphasised that the adoption of a technology-driven approach, such as artificial intelligence (AI), in the licensing process can equally help eliminate some of the bureaucratic bottlenecks.

Regarding human resources, participants revealed that the regulatory agencies had inadequate staffing levels, given the volumes of work they had to discharge. The inadequate staffing situation not only affects regulators’ ability to perform their administrative functions but also hinders them from conducting frequent compliance monitoring exercises in sand mining communities. The key informant at the EPA remarked:

Aside from addressing issues with sand mining, we also focus largely on illegal gold mining, which has gained national discourse and public outcry. We cannot be seen everywhere at the same time, given our current staffing levels and the extensive operational jurisdictions of our few staff [(EPA, 2021)].

The finding also revealed that the MINCOM and EPA were not administratively decentralised at the district level, thus operating either at the zonal or regional levels, respectively. Participants advocated decentralising the EPA and MINCOM to Ghana’s 261 administratively decentralised local government structures, emphasising the proximity of these regulatory bodies to mining communities as a strategic mechanism for effectively governing sand mining.

#### Information asymmetry on sand mining.

The interactions revealed that the regulatory agencies had inaccurate and insufficient information about the current state of sand mining in the study communities and other sand mining areas in Ghana. The agencies lacked a comprehensive database of sand miners and sand mining sites, which undermined their monitoring and enforcement capabilities. For instance, the key informants at both the EPA and MINCOM admitted they were unaware of the widespread illegal sand mining in certain areas of the Gomoa East district. This information gap undermines the implementation of appropriate strategies to address the negative effects of sand mining in the study areas.

#### Absence of bylaws for sand mining.

Local government authorities in Ghana have the legislative authority to enact bylaws as an additional legal framework for governing resource extraction within their jurisdictions. This includes permit requirements, rehabilitation standards, social standards, levies, and sanctions for noncompliance, among others. It was revealed that none of the local government authorities had passed bylaws on sand mining. Some of the participants expressed concern that not only had the local authorities failed to enact sand mining laws, but they were also inactive in enforcing the existing mining laws. The situation was particularly worrying because the majority of residents in the mining areas faced multidimensional insecurities stemming from illegal sand mining. The absence of bylaws on sand mining, therefore, raised concerns about the local authorities’ commitment to addressing widespread illegal sand mining in their areas.

## Discussions

Similar to the lack of policy responses to sand mining in many developing countries [9,40], Ghana’s sand mining sector is largely unregulated or poorly monitored. The inefficient application of the legal framework has culminated in widespread illegal sand mining and attendant adverse socio-ecological and economic externalities experienced by residents in the host communities. For instance, the acquisition of mining permits, which commit sand contractors to strict requirements, was largely not implemented. As a result, most sand miners operated without permits or used a single permit at several locations. The illegal sand mining was exacerbated by a weak culture of governance in compliance monitoring and enforcement.

The voracious sand mining without permits and licenses is attributed to a bureaucratic licencing process, inconsistent regulatory compliance monitoring, political patronage, and a weak sanctions regime. The finding is similar to that of Indonesia, where sand miners mostly used single permits for different sites [41]. Empirical evidence from Niger also confirms that local businesspersons and politicians facilitate their friends or cronies in engaging in mining activities without the required licences and permits [42]. Conversely, some European Union (EU) countries, such as the United Kingdom (UK), the Netherlands, and Germany, have efficient regulatory enforcement and compliance systems for sand mining [9,43]. These countries implement strict, well-enforced permit regimes in which Environmental Impact Assessments (EIAs) are strictly upheld [9,10], thus providing useful lessons for the Ghanaian regulatory and governance regime.

The study also highlighted the non-involvement of local stakeholders in the governance of sand mining, particularly in land allocation, licensing and permits, and monitoring and enforcement. Similarly, collaboration among regulatory agencies was inadequate, leading to administrative bottlenecks, insufficient information flow, inconsistencies across jurisdictions, erroneous interpretations of legal frameworks, and inter-agency conflicts. The finding corroborates the finding of Hausermann et al. (2018 [44]) that residents of mining communities are often not involved in or informed about proposed mining undertakings. Insufficient collaboration among stakeholders had created conditions for chiefs and clan heads to connive at illegal sand miners for their own monetary gain. According to Boafo et al. (2019 [45]), informal actors abandon their roles in regulating mining activities as a result of disregard by state regulatory actors. The inadequate involvement and information flow among state and non-state actors often result in unanticipated farmer displacement, which threatens land-based livelihood security [46]. Conversely, the adoption of interactive governance systems by the UK, the Netherlands, and Ireland has contributed to efficient compliance monitoring, mitigating the environmental risk of sand mining, and securing the well-being of residents in the mining areas [28].

Establishing regulatory structures capable of implementing the legal framework is crucial to the sustainable governance of sand mining. The study showed that both the EPA and MINCOM had inadequate human resources and state-of-the-art technology to effectively regulate the sand mining industry. The fact that sand miners operated from dispersed locations and in a nomadic manner further undermined regulators’ enforcement capacity. According to Adu-Baffour et al. (2021 [25]), inadequate human resources within regulatory agencies were responsible for widespread illegal mining in Ghana. Adoption of advanced monitoring systems and logistics, such as geographic information systems and drones, can enhance regulators’ enforcement capabilities and reduce the risks associated with monitoring sand miners, especially at night. Studies in Australia [47], the Netherlands [28], and the UK [9] concurred that adequate use of modern methods of compliance monitoring enhances effective governance of the sector.

The findings also showed that sand miners often made unilateral decisions regarding the compensation value for adversely affected persons, even though Act 703 stipulates the Land Valuations Division’s intervention to ensure commensurate compensation. The absence of adequate compensation for the majority of affected persons was due to most residents in the mining communities lacking tenure security or a legal right to farmland [14,48]. Family heads or local chiefs who held the land in trust for their families received large sums of money from the sand contractors, while those who lost their crops to the miners received almost no compensation. The inadequate application of public valuation estimates by regulatory agencies is consistent with findings in Zimbabwe [49] and Vietnam [50], where persons adversely affected by sand mining are not adequately compensated to pursue other livelihood activities.

The governance challenges underscore the weakness of the regulatory agencies in safeguarding the environment and the livelihoods of the residents in the sand mining communities. If regulatory agencies are constrained by inadequate human resources and logistics, information asymmetry, and limited stakeholder collaboration, ensuring sustainable sand mining becomes impossible. The consequence is that the residents would have to bear the negative outcomes of unsustainable sand mining. That notwithstanding, the SWOT analysis revealed an opportunity for the agencies to coordinate and collaborate through the sharing of information, logistics, and human resources for joint monitoring exercises. This will ensure efficient use of the few resources available to the agencies. The agencies could also work with the media to reduce the threat and the interference by ‘big men’ through media coverage of the issue.

## Conclusions and recommendations

Illegal sand mining is widespread in Ghana, with associated adverse social, economic, and environmental externalities. The industry is regulated by the broad institutional framework developed for Ghana’s entire mining industry. As a result, the existing governance structure is fragmented among several administrative agencies. Therefore, provisions in the existing laws, including the acquisition of permits and licences, the involvement of host communities, avenues for complaints, and compensation for negatively affected persons, are not adequately implemented by the regulators. The inadequate application of the laws was attributed to factors including insufficient human resources, lack of advanced monitoring technology, lack of collaboration among stakeholders, interference by government officials or big men, inter-institutional conflicts, and information asymmetry in sand mining.

Four recommendations are made to address the governance lapses embedded in the regulation of the sand mining industry. First, the Ministry of Lands and Natural Resources should lead the process to review outdated sections of the laws and align provisions across different laws relating to sand mining into a specific policy framework for Ghana’s sand mining industry. Amalgamation of the sand mining laws will help address the peculiar challenges inherent in the sector’s governance. Besides, it will curb the overlapping roles and inter-agency conflicts among the regulatory agencies. Second, the central government should adequately resource the regulatory agencies with advanced monitoring technology, logistics, and personnel to enable them to fulfill their mandate. The provision of modern, state-of-the-art technology and human resources will enhance enforcement and compliance systems, improve productivity, eliminate excessive bureaucratic delays in the licensing regime, and boost staff morale.

Third, the Ministry of Lands and Natural Resources should institute consultative mechanisms and collaboration among regulatory agencies through the sharing of information, joint compliance monitoring exercises, and involvement of residents, especially local opinion leaders, in sand mining decisions. Involvement of key local actors, such as assembly members, chiefs, and community members, will boost the enforcement capacities of formal state regulators by leveraging their rich knowledge of the local terrain. Besides, stakeholder involvement will ensure optimal use of the limited resources available to the regulatory agencies by eliminating uncoordinated and duplicated activities. Fourth, officials who interfere in the regulation of sand mining should be reported and adequately sanctioned to deter others. This will ensure that only entities that meet the legal requirements for restricted mining leases are granted permits. Overall, this study provides new insights into the application of the legal framework for sand mining, using two selected local government areas as case studies. It offers an innovative approach to enhancing collaboration among formal stakeholders and informal actors, the use of advanced monitoring technology, and the development of a sand mining policy to address the widespread and unsustainable sand mining in Ghana.
